# Development and validation of the Hospitality Axiological Scale for Humanization of Nursing Care

**DOI:** 10.1590/1518-8345.1767.2919

**Published:** 2017-08-03

**Authors:** José María Galán González-Serna, Soledad Ferreras-Mencia, Juan Manuel Arribas-Marín

**Affiliations:** 1 PhD, Researcher, Centro Universitario de Enfermería San Juan de Dios, Universidad de Sevilla, Bormujos, Sevilla, Spain.; 2 PhD, Full Professor, Escuela de Enfermería y Fisioterapia San Juan de Dios, Universidad Pontificia Comillas, Ciempozuelos, Madrid, Spain.

**Keywords:** Humanization of Assistance, User Embracement, Bioethics, Psychometric, Factor Analysis, Statistical, Nursing

## Abstract

**Objective:**

to develop and validate a scale to evaluate nursing attitudes in relation to hospitality for the humanization of nursing care. Participants: the sample consisted of 499 nursing professionals and undergraduate students of the final two years of the Bachelor of Science in Nursing program.

**Method:**

the instrument has been developed and validated to evaluate the ethical values related to hospitality using a methodological approach. Subsequently, a model was developed to measure the dimensions forming the construct hospitality.

**Results:**

the Axiological Hospitality Scale showed a high internal consistency, with Cronbach’s Alpha=0.901. The validation of the measuring instrument was performed using factorial, exploratory and confirmatory analysis techniques with high goodness of fit measures.

**Conclusions:**

the developed instrument showed an adequate validity and a high internal consistency. Based on the consistency of its psychometric properties, it is possible to affirm that the scale provides a reliable measurement of the hospitality. It was also possible to determine the dimensions or sources that embrace it: respect, responsibility, quality and transpersonal care.

## Introduction

Hospitality is an ethical value that guides the actions in the nursing profession in order to ensure an adequate care for patients, providing them quality care and comfort^1^. It is also an essential value for the adaptation of individuals to their stay in hospitals or in any area where health care is provided^2^. Hospitality or receptivity is empirically associated with humanization of care^3^ ^-^ ^4^. It implies an altruistic attitude based on humanitarian understanding of the practice of the nursing profession, which is committed to transpersonal care^5^.

It has been suggested that hospitality is influenced by several factors such as behavior, product and environment and, of these, those relate to behavior have been identified as the most important ones^6^. Hospitality or receptivity represents, in theoretical terms, significant advancement of humanization in health care, since providing hospitality attitudes is important for the curing process^7^. Implementation in the health care practice, both the ethics of hospitality of Derrida and the ethics of alterity proposed by Levinas, provide a possibility to increase the moral quality of the relationships between health professionals and patients^8^. However, there is still a need to clarify the theoretical construction of hospitality, so that it is conceptually defined in all its fullness. Therefore, the academic world should render its contribution to this process by carrying out studies on this subject and disseminating their results to society^9^.

The nursing model based on the tradition that comes from the figure of Saint John of God^10^ ^)^ goes beyond the understanding that other authors have had about receptivity or hospitality in the health sphere. This model emphasizes hospitality as a receptivity paradigm, which encompasses a set of sub-values necessary for a humanized patient care: respect, responsibility, quality and spirituality. The humanistic and anthropological philosophy of the Hospitaller Order of the Brothers of Saint John of God (OHSJD acronym in Spanish) has a key role in the value hospitality, because for them this term means alterity or humanization of the personal relationships of professionals and patients, as well as the social collectivity, i.e., the mutual concern for the each other. For the OHSJD, hospitality means receptivity, effective physical, moral, psychological and social support, valuing the multiple aspects of human needs^11^.

The culture of an institution is based on the values that are translated into reality of its functioning and dynamism. Previous studies have demonstrated how it is possible to estimate the axiological utility of the professional values of the codes of professional conduct in the health sphere through Likert-type scales^12^ ^-^ ^13^. The result of this estimation reports on the shared value system of a group and how it emphasizes values ahead of others and how the members of a group associate the different values expressing underlying axiological factors.

The objective of this work is to validate a scale in which nursing professionals and students consider the value hospitality or receptivity as a paradigmatic construct composed by underlying axiological factors in various dimensions or factors that characterize it.

## Method

### Participants and procedure

Before its beginning, the Institutional Committee on Bioethics of the OHSJD approved the study with opinion number 20101109a. The Axiological Hospitality Scale (AHS) was applied to 499 professionals from five Saint John of God Hospitals in central and southern Spain, in the cities of Seville, Malaga, Ciempozuelos (Madrid) and Santa Cruz de Tenerife. It was also applied to undergraduate Nursing students in two universities promoted by the OHSJD, the Comillas Pontifical University in Madrid and the University of Seville. Participants responded voluntarily and anonymously. In total, 52.6% were nursing professionals, 21.8% were third grade students and 25.6% were fourth grade nursing students. The average age of the participating professionals was 32.91 years, with age range from 22 to 58 years, and the average age of the students was 24.11 years, with age range from 20 to 46 years. In terms of gender, 402 were women (77.5%) and 117 men (22.5%). Participants responded voluntarily in the period from 2011 to 2015. 

### Development of the instrument

The proposal of items was developed based on a review of the theoretical foundation of the construct and specific instruments for the measurement of values related to hospitality. It was referred to a focus group of experts. The resulting prototype, composed of 30 items, was applied to the study sample. In this version, the indicators were evaluated using a 7-point Likert-type scale, where 0 indicates “no importance” and 7 indicates “maximum importance”. Based on the analysis of the data and results obtained, the study of the reliability and validity of the scale was carried out.

### Analysis

The validation of the measuring instrument was carried out, in a first stage, by techniques of reliability analysis and factorial analysis of principal components. Those indicators that conceptually best fit the theoretical meanings of the proposed construct were selected. Subsequently, a first-order Exploratory Factor Analysis (EFA) was carried out to identify the possible dimensions that were conceptually implicit in the construct. Considering the resulting dimensions of the EFA and the theoretical framework of the study, two models for its Confirmatory Factor Analysis (CFA) were proposed using *Structural Equation Modeling* (SEM) techniques. To verify the goodness of fit and the validity of the models, it was considered the results both the χ^2^ test and the descriptive goodness of fit measures. The software used for the EFA was the IBM SPSS *Statistics* for Windows version 20.0 (IBM Corporation, New York, NY, USA). The software EQS 6.2 for Windows was used for the CFA of the model^14^. The several goodness of fit and residual measures were calculated by the Maximum Likelihood Robust Estimation method^15^, as they are less sensitive to the absence of multivariate normality (Mardia’s Coefficient > 5) and show the distributions of the obtained data.

## Results

### Reliability and exploratory factor analysis (EFA) 

In the EFA carried out on the final prototype of the 30-item scale, it was observed that the most significant indicators were grouped in four dimensions, resulting in a 17-items scale after selecting those that showed the highest scores in each of the different factors.

The resulting 17 items and their meaning are:

-Receptivity (Friendly treatment or hospitality offered by the professional to the user)
-Personalized comprehensive care (Assistance that provides global, biopsychosocial and spiritual care to the particular needs of each person)
-Altruism (Commitment in the pursuit of the patient’s good, even at the expense of his own good, but without nullifying itself)
-Professional autonomy (Quality of the professional who, for certain tasks, does not depend on anyone)
-Scientific quality (Scientific importance and excellence. It corresponds to what science knows)
-Proximity (Emotional closeness, friendly treatment)
-Compassion (Feeling of solidarity and concern for those who suffer difficulties or misfortunes)
-Competence (Competence, ability, suitability to perform the actions of the health professional)
-Scientific knowledge (Have data and adequate scientific understanding on health issues)
-Diligence (Promptness, agility, haste, attention and agility in providing care)
-Empathy (Psychological and affective identification of one person with the emotional state of another)
-Justice (Give each one what corresponds or belongs to them)
-Prudence (Discern and distinguish what is good or bad, to follow or avoid it. Sense, good clinical judgment)
-Respect for life (Consideration and deference to life. No maleficence)
-Respect for the autonomy of the users (Consideration and deference to the desires, values and beliefs of users)
-Simplicity (Work naturally, with spontaneity, plainness)
-Veracity (A way of expressing oneself free of pretenses. Always tell the truth)

The scale showed a high internal consistency index, with a Cronbach’s Alpha value=0.901. In the EFA of the scale, the Kaiser-Meyer-Olkin (KMO) measure of “sample adequacy” showed a value of 0.931 (close to unity), the Bartlett sphericity test (p<0.001), and the χ2 value=3213.58 (df=136). 

Table 1 shows the results of the EFA (by the principal components method and the Promax rotation) computed for the responses of the questionnaire (values less than 0.30 were removed to facilitate reading) and there were 4 main components identified in the extraction, which explain 59.528% of the total variance. Rotation reveals the existence of a factorial structure in which the indicators are grouped into 4 components. There liability analysis of the subscales including the indicators of the four factors confirmed that they have good internal consistency indexes with values of Cronbach’s α varying from 0.70 to 0.80. These indexes are considered as adequate since the number of indicators for each factor is reduced. The homogeneity indexes were also satisfactory, with item-total correlations above 0.43 in each indicator, with values above 0.30 being considered acceptable^16^. Therefore, the proposed indicators allowed finding differences among the subjects in relation to the factors resulting from this study. 

**Table 1:** Exploratory factor analysis of the Axiological Hospitality Scale (AHS). Configuration matrix. Factorial loads, explained variance and Cronbach’s Alpha (N=499).Seville, Malaga, Ciempozuelos (Madrid) and Santa Cruz de Tenerife, Spain, 2011-2015

					**Components**				
			**X**	**σ**	**1**	**2**	**3**	**4**	**Explained Variance**
**Responsibility**	α **= 0,772**	**Personalized comprehensive care**	**6.23**	**1.045**	**0.891**				**40.44%**
		**Receptivity**	**6.31**	**0.967**	**0.768**				
		**Empathy**	**6.09**	**1.096**	**0.624**				
		**Proximity**	**6.02**	**1.079**	**0.490**		**0.403**		
**Respect**	α **= 0,799**	**Veracity**	**5.59**	**1.339**	**-0.307**	**0.906**			**6.93%**
		**Justice**	**5.89**	**1.354**		**0.628**			
		**Respect Autonomía**	**6**	**1.143**	**0.338**	**0.623**			
		**Respect for Life Vida**	**6.32**	**0.987**	**0.453**	**0.568**			
		**Prudence**	**5.87**	**1.125**		**0.516**			
**Transpersonal care**	α **= 0,704**	**Altruism**	**4.89**	**1.649**			**0.772**		**6.54%**
		**Compassion**	**5.38**	**1.479**			**0.728**		
		**Simplicity**	**5.44**	**1.464**		**0.527**	**0.549**		
		**Diligence**	**5.45**	**1.390**			**0.516**		
**Quality**	α **= 0,742**	**Quality**	**5.77**	**1.323**				**0.916**	**5.61%**
		**Knowledge**	**5.98**	**1.135**				**0.750**	
		**Autonomy**	**5.52**	**1.330**				**0.388**	
		**Competence**	**6.06**	**1.135**	**0.311**			**0.367**	
**Cronbach’s Alpha = 0.901**			**Total Explained Variance**				**59.53%**		

Extraction Method: Principal Component Analysis.

Rotation Method: Promax Normalization with Kaiser.

Rotation has converged into 8 iterations.

Based on these results, the resulting latent variables were operationalized according to the observable variables. This has allowed affirming that the construct Hospitality can be structurally configured in four components or dimensions: a) “RESPECT”; b) “RESPONSIBILITY”; c) “QUALITY”; and d) “TRANSPERSONAL CARE”. The Respect dimension is made of values representing respect for life, for the autonomy of the user and for fair treatment. The Responsibility dimension consists of values representing acceptance of the user’s closer personalized care. The Quality dimension involves values representing nursing actions based on competence and professional autonomy as well as a general and broad concept of quality that encompasses other structural or procedural elements. The Transpersonal Care dimension includes values representing the capacity of personal projection towards the user, with an altruistic motivation and a diligent care. All resulting factors showed significant intercorrelations.

The results of the second order EFA reflected a unidimensional factorial structure (Table 2). Consequently, a second order factor emerged as a factorial synthesis of the twenty-three indicators, which explained 44.7% of the variance, and was theoretically interpreted as construct “Hospitality”.

**Table 2:** Second order confirmatory factor analysis of the construct Hospitality. Configuration Matrix and Correlation Matrix. Factors of the AHS Scale. (N=499). Seville, Malaga, Ciempozuelos (Madrid) and Santa Cruz de Tenerife, Spain, 2011-2015

	**Component**	**Factor 1**	**Factor 2**	**Factor 3**	**Factor 4**
**Respect**	**0.860**				
**Responsibility**	**0.849**	0.664*			
**Quality**	**0.823**	0.612*	0.588*		
**Transpersonal care**	**0.815**	0.591*	0.584*	0.563*	

*Correlation is significant at the 0.01 level (bilateral).

Extraction method: Principal component analysis.

Rotation method: Promax with Kaiser Normalization. 1 component extracted.

### Confirmatory factor analysis

In order to confirm the underlying structure, two rival measurement models that were plausible from the theoretical and empirical point of view were used. The 4-factor model represents the most satisfactory fit indexes. The Satorra-Bentler scaled χ2 showedanS-B χ^2^value=241.95 (df=113, p<0.00000). Regarding the likelihood estimation of the model, the normalized Chi-square value (2.14) was within the recommended levels^17^. Regarding fit indexes, the Normalized Fit Index (NFI) showed a value of 0.900, the Non-Normed Fit Index (NNFI) showed a value of 0.932 and the Comparative Fit Index (CFI)^18^ ^)^ showed a value of 0.944. The value of the Root Mean Square Error of Approximation (RMSEA)^19^ was 0.048, all indicating a satisfactory fit with values ranging from 0.9 to 1^20^. It can be concluded that all goodness of fit indexes calculated show an acceptable fit between the postulated theoretical model and the sample data, so it has not been possible to prove that the model is incorrect and it has been proven to be one of the possible acceptable models^21^.

In a more detailed analysis of the values, which resulted in the standardized solution for the proposed model (Figure 1), it was observed that all parameters have positive and significant estimates.

**Figure 2:**
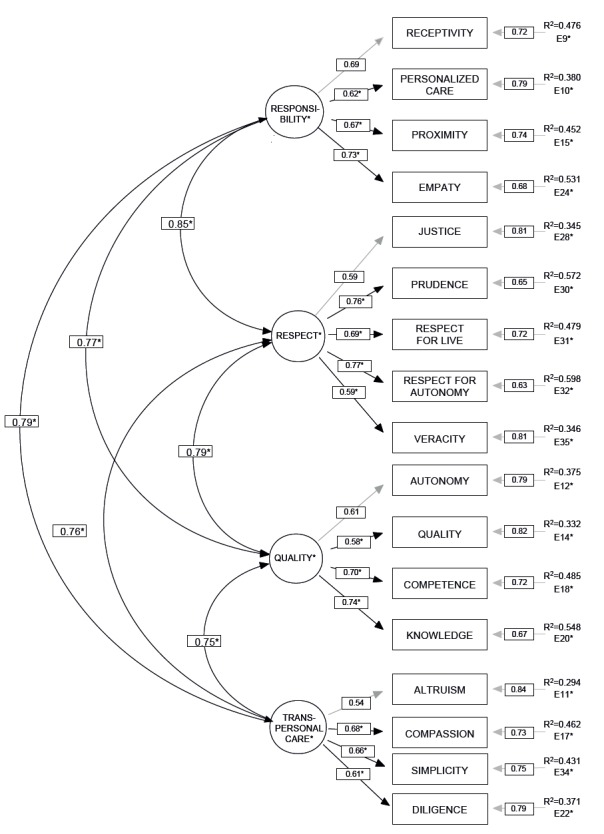
Standardized solution of the parameters estimated for the measurement model Axiological Hospitality Scale (AHS) (N=499)

The indicators show an adequate reliability, with factor loads higher than 0.50 and R^2^values higher than 0.30, except for the item “altruism”, which shows R^2^=0.294, very close to the recommended minimum. Composite reliability was estimated for each construct with values ranging from 0.71 to 0.81, above the recommended minimum^21^. 

Regarding the convergent validity of the constructs, the mean variance estimated from the first order factors assumed values ranging from 0.39 to 0.68.

Finally, it was verified that the root mean square calculated for each construct showed a value higher than the correlation presented by each one of them in relation to all the other ones, which evidences the discriminant validity^22^.

The following step was to test the model in a random sample with half the study participants. The goodness of fit measures from the data of this random sample was considered acceptable with an S-B χ2=171.76 (df=113, p<0.00003). The absolute fit index (RMSEA) showed a value of 0.046, within the accepted fit range, and the likelihood evaluation of the model showed a normalized χ2 with a value of 1.52, also within the recommended levels. Regarding the incremental fit indexes, the Normed Fit Index (NFI) showed a value of 0.865, the Non-Normed Fit Index (NNFI) showed a value of0.938 and the Comparative Fit Index (CFI) showed a value of0.948. All these indexes showed acceptable values and similar to those calculated for the whole sample.

## Discussion

The improvement of the practices of receptivity or hospitality are now a challenge for health services^23^. Nursing professionals are able to identify how the reception should be carried out through qualified listening, humanization, responsibility and commitment to the needs of the other. However, in practice, so that these actions are recognized as nursing care, the nurses should focus on relational care^24^.Hospitality generates attitudes based on professional values^25^, as it is capable of promoting the relational bond between professionals and users, allowing to stimulate personal care, improve understanding of the disease and promote co-responsibility during treatment. It also enhances universal access, strengthens multidisciplinary and intersectional work, qualifies care, humanizes practices and encourages actions aiming to combat injurious^26^.The evaluation of the axiological estimate of hospitality is important to know the attitude of nurses in relation to this central value in the practice of health care. Organizational culture serves as reference for the members of an organization and provides guidance on how people should behave in it. The culture of hospitality consists of a collective experience within the OHSJD, involving values that represent it. It is necessary to remember that ethical values are the organizational and fundamental basis of every society, profession and person. They give meaning and identity to the professional group. They have a strong motivating component and represent an important indicator of the quality of care, humanization, patient satisfaction and the professionals themselves. Properly developed professional values guide clinical practice according to professional ethics^27^.

In the literature, no other scales to estimate the construct Hospitality or receptivity were found since the scale proposed here is an original and innovative contribution in this field of study, which can be applied to the development of the organizational culture in order to promote humanization of nursing care and health improvement.

Although an exact equivalence between the values selected for the scale and those professed by the OHSJD has not been achieved, the values of each dimension of the scale adequately represent those values explicitly professed by the OHSJD^28^.

One limitation of the study is that of the four declared values, spirituality is the least represented value in the AHS. However, spirituality is included in the AHS from the perspective of Transpersonal Care, which ultimately presupposes a transcendent approach of a professional activity that is not focused on itself but on the patient and family. This projection is an expression of otherness and has a spiritual meaning in that it is transcendent. In fact, the spiritual care of the patient and his family has as fundamental expression the transpersonal care performed by the health professionals^29^.

A second limitation of this scale is that the sample used to carry out the validation consists of professionals and students of an institution with a culture of values that incorporates in its tradition the value hospitality and gives it an internal validity. In order to corroborate the external validity of the scale, it would be necessary to extend this study to samples from other organizations outside the OHSJD context, in order to verify that validity.

## Conclusion

In this study, it was analyzed the development of an instrument that allows measuring the behavior in relation to the construct Hospitality according to the perception of the nursing professionals and students in the context studied.

The scale showed a high internal consistency index (0.901) and the subscales showed reliability coefficients higher than 0.70. Validation using the EFA and CFA methods has allowed to confirm the factorial structure of the scale and to demonstrate its validity. The results obtained in the CFA allow postulating that the construct Hospitality encloses four dimensions: “Respect”; “Responsibility”; “Quality” and “Transpersonal Care”. 

Regarding the psychometric characteristics of the Axiological Hospitality Scale, it has been possible to confirm its factorial structure by a measurement model that has shown satisfactory goodness of fit measures, so that it can be affirmed that the scale has allowed evaluating the perception of Hospitality with an appropriate level of reliability and validity.

These results corroborate the usefulness of this tool considering the scarcity of instruments to evaluate the construct hospitality in nursing professionals and students.
